# Community mobilization and policy demands in the context of the COVID-19 pandemic: A transnational dialogic non-extractive research approach

**DOI:** 10.1371/journal.pgph.0006384

**Published:** 2026-10-22

**Authors:** Ruth Iguiñiz-Romero, Pamella Liz Nunes Pereira, Christiane Falge, Stephanie Mayell, Mylène Fauvel, Theresa Martens, Luiza Witzel Farias, Lucero Diaz Gomez, Paulo Trivellato, Franziska Satzinger, Deisy de Freitas Lima Ventura, Michael Knipper, Laurence Monnais, Anne-Emanuelle Birn

**Affiliations:** 1 School of Public Health and Administration, Universidad Peruana Cayetano Heredia, Lima, Peru; 2 School of Public Health, University of São Paulo, São Paulo, Brazil; 3 Department of Community Health, Hochschule für Gesundheit University of Applied Sciences Bochum, Bochum, Germany; 4 Department of Anthropology, University of Toronto, Toronto, Canada; 5 Department of Social Work, Université du Québec en Outaouais, Gatineau, Canada; 6 Institute for Critical Urban Geography, University of Münster, Münster, Germany; 7 Institute of International Relations, University of São Paulo, São Paulo, Brazil; 8 Brazil Institute, King’s College London, London, United Kingdom; 9 Department of Health Sciences, Fulda University of Applied Sciences, Fulda, Germany; 10 Conselho Nacional de Desenvolvimento Científico e Tecnológico (CNPq) Research Productivity Fellow – Level 1A, Brasilia, Brazil; 11 Institute of the History, Theory and Ethics of Medicine, University of Giessen, Giessen, Germany; 12 Institute of Medical Humanities, Université de Lausanne - Centre Hospitalier Universitaire Vaudois (CHUV), Lausanne, Switzerland; 13 Department of Global Development Studies, University of Toronto, Toronto, Canada; University of Washington Seattle Campus: University of Washington, UNITED STATES OF AMERICA

## Abstract

This essay discusses the interplay of epistemological-methodological approaches and research practices developed within a global health study that aims to understand and strengthen community actions and organizing by – and preserve the memories of – made-marginalized places and people during the COVID-19 pandemic. The project has brought together community actors and social justice scholar-advocates in two Global South countries (Brazil and Peru) and two Global North countries (Canada and Germany) to explore and dialogue around how community mobilization efforts during the pandemic sought to influence policymaking toward health equity and social justice. Rather than emphasizing our research results per se, we relay our experiences of countering dominant, asymmetrical approaches to global health research, both in theory and practice, based on a shared belief that community engagement is central to societal transformation. We provide an in-depth account of *how* we have carried out our project – through what we aspirationally name Dialogical and Non-extractivist Approaches (DNA) – using an analytic narration of the possibilities of global health research anchored in an ethical dialogic praxis, guided by principles of non-extractivism and Freirean action research.

## Introduction

Policy responses to COVID-19 have been widely characterized and assessed in terms of national responses and international comparisons therein [1,2]. These dominant analyses underplay two vital dimensions of pandemic responses: subnational political levels, which host crucial public health and social policymaking bodies in many countries; and the role of community mobilization and solidarity actions.

During the pandemic’s initial phases in 2020, many community activities were celebrated, often sentimentally, as features of “human resilience” and “social capital” [3]; by contrast, we argue that these depictions are highly problematic. Indeed, such accounts downplay the struggles and commitment of social actors from communities around the world who have been made-marginalized^i^ [4] through historical processes of colonialism, ongoing imperialism, and the hegemony of contemporary global capitalism. Meanwhile, concerted and creative mutual aid strategies undertaken in the absence of adequate government responses tend to be minimized by mainstream accounts [5,6], which are informed by dominant neoliberal and neocolonial (in the case of North-South development discourse) policy approaches. Importantly, in contexts marked by informal jobs, low incomes, inadequate services, unsafe housing, and barriers to rights, countless historically made-marginalized communities self-organized during the pandemic in order to secure shelter, food, medical care, and income [7,8].

Our collective research endeavor (Social Mobilization as Policymaking Lever – SMAPL) addresses the silences around such community efforts: we explore how place-based social justice actions and community resistance to address the crises spawned and exacerbated by the COVID-19 pandemic have unfolded and interacted with decentralized policymaking^ii^  in six settings across four countries in the Global South^iii^ [9] (Brazil, Peru) and North (Canada, Germany). Utilizing dialogic and collaborative knowledge-building methods, we have worked with local actors – both residents and those who are supporting them – in made-marginalized and racialized neighborhoods in São Paulo and Montréal, Indigenous Awajún communities in the Peruvian Amazon, culturally diverse areas within the German cities of Giessen and Bochum, and hubs of migrant agricultural workers (MAWs) in Southwest Ontario, to understand and learn from the range of their experiences in response to the common challenges of the pandemic (see Box 1).

**Box 1. Descriptions of the SMAPL Cases**

The Brazil case centered^a^ on community responses to the COVID-19 pandemic in **Jardim Colombo**, a favela in São Paulo whose majority Black population has experienced longstanding racial discrimination and exclusion linked to historical enslavement and contemporary spatial segregation, combined with state neglect and a precarious informal labor force. In Jardim Colombo, engaged community members and *solidaire* scholars created a repository of people’s narratives in video [10] and comic book [11] formats. In this way, the voices and complex experiences of residents who mobilized collectively during the pandemic for their families’ and neighbors’ survival are preserved as a form of community memory of resistance that belongs to the people for their own purposes.

In **Peru’s Amazonas region**, mobilizing by Indigenous Awajún communities was deeply informed by the accumulated experiences of organization [12] and resistance in the face of historical and continuing threats to their very survival. Also crucial, as the SMAPL team underlined, were a small set of sustained relationships of trust that had previously been established between community leaders and health personnel. The integration of knowledge [13] from traditional healers and knowledge-bearers with guidelines developed by local health personnel, some of whom are Indigenous, trained in intercultural medicine made it possible to provide care within the communities during the most critical moments of the pandemic. This knowledge integration also helped to dispel the enormous, and justified, suspicion of government-sanctioned vaccination campaigns.

From the outset, the government of the Canadian province of Québec relied on community organizations to address the emerging socio-economic needs resulting from both the pandemic and from public health guidelines [14]. Even as many of the public health measures further marginalized the most vulnerable communities, local initiatives in **Montréal** fostered “community-driven care” and mutual aid networks to counteract the harmful effects of the provincial response to the spread of SARS-CoV2. The bottom-up care responses highlighted by SMAPL researchers spanned food banks, mobile vaccination clinics, and community-led discussion groups by and for the most vulnerable groups (not least those with precarious migration status), especially in the multi-ethnic neighborhoods that were hit the hardest, including Parc-Extension, Montréal-Nord, and Côte-des-Neiges [15].

Also in Canada, organizers and advocates (including SMAPL team members) working with Central American and Caribbean migrant agricultural workers (MAWs) in **Southwest Ontario** –whose labor and living conditions have been likened to slavery [16] by Jamaican MAWs themselves– built on decades of mobilization and social solidarity to address both pandemic-related needs and longstanding harms. Organizers were engaged in multiple strategies (petitions, testimonials, demonstrations, legal cases, investigative media coverage, etc.) to draw public attention to MAWs’ struggles for adequate housing, fair labor conditions, and access to workers’ compensation, and to demand permanent resident status on arrival for all MAWs, as well as an end to closed work permits [17].

In the small German cities of Giessen and Bochum, community researchers and scholar-activists co-constructed collaborative spaces to discuss counterhegemonic perspectives on the pandemic and to support immigrant community members in recognizing and harnessing their own citizens’ and residents’ rights [18]. **Giessen’**s community researchers live in post-WWII working-class housing in the Nordstadt neighborhood (approximately 10,000 inhabitants): most come from low-income families, many with migration histories and who are affected by structural racism.

In **Bochum,** a post-industrial city in the Ruhr area shaped by longstanding labor and refugee migration, the SMAPL project and ongoing research-action work focuses on a deprived high-rise neighborhood (inner Hustadt, approximately 3,000 inhabitants) with limited local services and poor public transit access. Most residents live in low-income households; many are first- and second-generation refugees from Turkey (mainly Kurds), Afghanistan, Lebanon, Syria, and Iran. In both cities, community researchers identified manifold and compounding shortcomings of the state’s pandemic response vis-à-vis their own experiences, linking these problems to broader structural issues, in Bochum responding by initiating a community-based vaccination campaign [19].

^a^ Because the formal Trans-Atlantic Platform funding for this project has now ended, we use the past tense when discussing certain activities that took place during the study period. However, because our research commitment and collaborations with the communities, and within and across teams, is continuing, we use the present progressive tense and gerunds to describe our ongoing engagement.

Although our project comprises multiple teams and locales, and is funded by research agencies from four nations, it does not heed a typical global health partnership arrangement in which well‑resourced institutions in the Global North unilaterally set priorities, and then select Global South partners to maximize project outputs and visibility. By contrast, our research endeavor has sought to be horizontal, dialogical, and South-North-driven. This is in marked contradistinction to prevailing North-to-South global health “collaborations,” wherein partners in the Global South mainly implement activities and supply local access and data, often with limited influence over agenda-setting, governance, and benefit-sharing. Moreover, our collaborations have arisen from existing *non-formal* relationships with communities and among the researchers –academic- and community-based alike– both within and between country teams. It is bottom-up in the sense that the research questions, approach, and “products” respond to the needs and preoccupations articulated by the communities with whom we work. Our project’s primary aim is to support transformational change, even as we recognize that the structural impediments remain massive. Yet we draw hope from the possibility, at times the reality, that political and power structures can be fragile and malleable, enabling us to locate the weak joints of, and thus work to dismantle, oppressive societal architecture.

As researchers from a variety of colonial, colonizing, and colonized contexts and legacies, we recognize the power asymmetries arising from these circumstances, including within and across our research teams. In responding to and addressing these concerns, we are committed to a research praxis that raises the voices of made-marginalized groups [4] within our communities and countries, as well as globally. In recognizing the contradictions and power imbalances of our own positionalities and trajectories – the PIs are tenured professors from privileged backgrounds, half based in the South, half in the North, who all studied in North American and European universities; the students and trainees come from a mix of working-class and middle-class backgrounds, and various are racialized and are from made-marginalized families and communities – we seek to contest the status quo of academic hierarchies and research/knowledge diffusion practices. Additionally, most of the Global North-based researchers have experience living, working, and studying in the South; conversely, the SMAPL project has enabled a growing number of South-North exchanges, especially for trainees, further nourishing the multidirectional and horizontal learning within and across our research teams [20].

Our global health project rests on the incorporation of what we have aspirationally, if ironically, named as dialogical and non-extractivist approaches (DNA) into the very philosophy and design of this endeavor, not as an afterthought or “fix-it effort.” To this end, we distance ourselves from mainstream discourses in order to define and re-appropriate the concept of global health as a field of engaged activist research, South-North collaboration, and community-academic learning. In this spirit, the research questions we pose(d) employ(ed) a DNA lens from the outset. In accordance with Paulo Freire’s conceptualization (see ahead), dialogical praxis refers to a methodological and epistemological framework that emphasizes mutual recognition, reciprocity, and horizontality in the production of knowledge and interaction with communities. This framework is aimed at transforming reality through a continuous process of critical reflection and action. A non-extractivist research approach seeks to foreground the dignity and agency of the individuals and collectives involved in the project. We adhere to the belief that researchers should not treat participants as mere objects of study, but instead enable their voices and experiences to emerge authentically, and in all their complexity.

Putting into practice the approach described above, this essay provides an analytic narration of how global health research anchored in DNA might be conducted both ethically and in as non-hierarchical manner as possible. Based on an in-depth account of *how* we have carried out our project, we describe how we tackled these challenges both amid the dynamics of our global research team, and between each country research team and community members and participants. Corresponding to the article’s sub-sections, we pay particular attention to three interrelated dimensions of *doing* our research: 1) the theoretical-epistemological; 2) the structural; and 3) the methodological. We end by reflecting on how DNA applied to global health research might become transformational.

## Multiple voices naming the world together: Our theoretical-epistemological framework

Our DNA research methodology is framed by our commitment to prioritizing theoretical and scholarly references from the Global South,^iv^ especially thinkers who critically discuss the research process, starting with the social position of researchers themselves. To guide us in this reflexive thinking, *both* in terms of bringing new perspectives to –and ways of engaging in– global health research *and* in our collective and individual learning and unlearning journeys, we take the work of Brazilian educator-philosopher Paulo Freire [21,22] as the central axis of our research across all cases.

A foundational thinker in critical pedagogy, Freire’s most important contribution is/was in Popular Education. Emerging from adult literacy efforts and experiences, it was soon taken up by various social movements and Brazil’s universal health system, among other societal sectors. Popular Education unleashes pathways guided by love, affection, and joy; here, the knowledge and experiences of individuals and groups (collectives) are centered, grounded in cultural diversity, and emphasize a critical-reflective approach with emancipatory intent [23], that is, with the aim of countering and transforming oppressive and exploitative relationships (see further details below).

We also engage with the ideas of Donna Haraway [24], Veena Das [25], Gayatri Spivak [26], and Silvia Rivera Cusicanqui [27,28] to examine how we –as researchers writ large, as (mostly) women, and as people from different class, racial-ethnic, cultural-religious, migration, and national/regional backgrounds– (should) see and understand our own roles and power dynamics. These thinkers, in turn, align with various intellectual traditions, including critical race theory and racial capitalism [29], Third World Feminism [30], and Indigenous historical and political thought [31], which additionally shape our perspectives.

To prevent favoring our individual and collective researcher knowledge and worldviews, or potentially forcing these upon communities where we are working, we look to Freire’s [22] concept of “dialogue” as essential to avoiding what he calls “cultural invasion.” Accordingly, dialogue needs to be “the encounter between men [sic], mediated by the world, in order to name the world” [22, p. 88]. Transcending Karl Marx’s call to understanding the world as the path to transforming it [32], Freire believed that human needs are fulfilled through the very process of “naming the world” via dialogue to achieve a shared sense of the world and people’s role in remaking it.

Complementing Freirean dialogue, Das [25] and Spivak [26] emphasize the importance of recognizing the agency of those affected by distinct forms of violence, including “epistemic violence” unleashed by violent knowledge systems that silence and/or distort the expressions and experiences of subaltern groups. These authors advocate for research that allows “the oppressed” (even as Spivak critiques such homogenizing language) to express and name their experiences and recognize that they themselves are knowledge-bearers. This notion is in harmony with Freire’s *Pedagogy of the Oppressed* [22], which proposes that knowledge should be co-constructed through dialogue, not imposed by an external authority, to ensure that research is transformative as opposed to exploitative. Fundamentally, this approach values the agency of participants, positioning them as active knowledge and research contributors rather than passive subjects [22].

All three scholars highlight the need for forms of engagement that respect the autonomy and dignity of made-marginalized people, and urge for ethical research practices. Taking this approach requires that we, as researchers, acknowledge and interrogate how our own backgrounds, trajectories, prior experiences, beliefs, and assumptions variously influence the research process. For this reason, our positionality and reflexivity discussions are not confined to one section of the paper, but instead are interwoven as they appear relevant, at different moments.

Our research teams have actively engaged in dialogue with the various social actors in each site; our own communication within and across teams is also dialogic. Complementing Freire’s dialogic approach, we apply Haraway’s [24] “situated knowledge” to the context of knowledge co-production, which, taking on Spivak’s challenge [26], enables the subaltern (or made-marginalized or oppressed person/people) to be heard despite the “reign” of certain knowledge hegemonies. The classical approach to objectivity perpetuates the myth that it is possible to *see* something from the outside: making purely detached observations without having any influence on the phenomenon itself. Haraway [33] calls this disembodied vision “the divine trick.” In a similar vein, Rivera Cusicanqui argues that “true objectivity can only be achieved through an embodied, experiential engagement with the world,” emphasizing that knowledge must be rooted in lived realities rather than detached abstractions [34].

Taken seriously, these ideas prevent researchers from assuming that they can theorize all aspects of an event or experience. To address this challenge, we deployed Haraway’s notion of positionality to acknowledge the ways social and cultural positions affect the perspectives from which individuals and communities view and experience the world.

Compiling these theoretical and epistemological insights from mostly Global South thinkers, our ongoing analysis rests on a set of organizing dimensions, approaches, and concepts that traverse all cases, albeit unfolding differently in each setting [Table 1].

**Table 1:** Using DNA to think from/with the South: Analytical Dimensions/Approaches/Concepts.

Dimension/Approach/ Concept	Description	Illustrations/Reflections vis-à-vis our project
Historical Memory and Reading the World (Leitura do mundo) (Freire) [35] and Situated Communities (Ch´ixi) (Rivera Cusicanqui) [28,34]	Highlights the active presence of the past in “reading” the present; contemporary experience reflects continuation of long-standing colonial and structural violence. Seeks to identify contradictions and complexities in how Indigenous peoples, descendants of enslaved Africans, and racialized internal and international migrants live alongside, and in friction with, contemporary realities, recognizing that these tensions are multidirectional.	Understanding communities’ own characterizations of their identities and contexts as multiple, complex, and dynamic (i.e., not fixed, static, or pre-determined). Acknowledging practices that are simultaneously fully “modern” and profoundly rooted in “origin” or “traditional” lifeways. Linking the pandemic to past historical injustices, and/or mobilizing ancestral memory and practices to survive modern crises.
Situated Knowledge and Positionality (Haraway) [24]	Examines what counts as valid knowledge in terms of where/how knowledge is produced (situated/local knowledge vs. “objective/biomedical” models). Acknowledges how people’s social and cultural positions affect the perspective from which individuals and communities view and experience the world.	Recognizing explanations of the virus, contagion, or healing approaches using local frameworks alongside official medical discourse. Portraying each case in our project by taking into consideration how those made-marginalized define(d) the situation themselves. Reflecting on researchers’ own roles in generating data and interpretations, acknowledging how our own social and cultural positions shape the research process, both influencing and being influenced by local dynamics.
Discursive Power, Epistemic Violence, and the Politics of Voice (Spivak) [26] and (Freire) [22]	Analyzes the discursive traps that silence made-marginalized communities. Focuses on who is allowed to speak, and who is spoken for within overall structures of representation. Spivak calls for subaltern (or made-marginalized) people to be heard, despite the “reign” of certain (dominant) knowledge hegemonies, cautioning against homogeneous forms of representation.	Acknowledging situations in which participants explicitly contest or correct (and in some cases amplify) state or mainstream media narratives, asserting their own voices as the authoritative record. Seeing how critiques of local and global power structures emerge across communities, highlighting systemic inequalities and injustices in pandemic responses.
Everyday Materiality, Vulnerability & the Body (Das) [25] and (Freire) [22]	Moves away from macro-level structural narratives to focus on the micro-level of daily survival, pain, and bodily experience.	Validating descriptions of bodily suffering, hunger, domestic caregiving, solidarity networks, community aid, and the daily logistics of surviving lockdowns.
Dialogic Praxis (Freire) [21,22]	Plays out on distinct levels: across and within research teams and between researchers and communities. In rejecting the idea that only specialists and scholars are competent to know, speak authoritatively, and make decisions, serves as a prerequisite for horizontal relations and communications, which in turn create a bona fide sense of collaboration. Emphasizes the need for all parties to articulate their understandings, hopes, and dreams explicitly and unconstrained, thereby ensuring a meaningful exchange of perspectives.	Fomenting horizontality through researcher interactions and relations within and across country teams, and between Global South and North; seeking to break with academic hierarchies and typical North-South unidirectional dominance of global health partnerships in defining research agendas, theoretical frames/referents, and methodological approaches. Centering community articulations of political demands, forms of organizing, and identifying “root” causes and structures as targets for policy and societal change. Within each context, prioritizing experiences and aspirations of community organizers and integrating these perspectives into the research.
Mutual Learning, Co-production/shared Knowledge Production (Freire) [22]	Involves people learning from each other and creating knowledge together –mediated by their contexts–, “a process in which all grow and … arguments based on “authority” are no longer valid. Here, no one teaches another, nor is anyone self-taught.” [22 p. 80] Underscores that knowledge and knowledge production are not “owned” but shared.	Community researchers and organizers deeply engaging in and contributing to research design, implementation, analysis, and knowledge-sharing across all SMAPL sites (i.e., via a non-extractive process). “Giving back” outputs and research results to communities, who co-produce the knowledge, for their own use and purposes.
Community-led Social Justice Transformation (Freire) [22] and (Haraway) [24]	Emphasizes that academic research does not exist outside the world, but is in fact part of it and influences it. Recognizes, therefore, that all research is political and plays a role in social transformation; moves from remembering (reflection) to demanding systemic change (action). Embraces research-action and awareness-raising, thereby developing a critical consciousness (conscientização).	Involving continuous self-reflection and open dialogue about privilege(s) and hierarchies within our SMAPL team(s). Usurping hegemonic sites of power through (non-English) language choices, construction of project ideas, and methodologies “from below.”

Per Freire [22], dialogue comprises five interrelated aspects: love, humility, faith, hope, and critical thinking. For Freire, *love* is an ethical, committed concern for others that cannot exist in relations of domination; it is both the foundation of dialogue and dialogue itself. *Humility* necessitates recognizing the limits of one’s own knowledge and learning from others, and is needed to constantly re-create and collectively rename our world. *Faith* in people involves trusting their capacity to interpret their reality and act accordingly, leading to relationships based on mutual trust. *Hope* is rooted in the never-ending search for fulfillment, which can only unfold in communion with others. Although such completeness can never be achieved, it is essential to starting and sustaining dialogue as a transformative practice.

Lastly, Freire emphasizes the importance of *critical thinking*, the shared practice of reflecting on reality and questioning one’s own assumptions, that is, to dialogue. Within this fifth principle, three elements are highlighted: dialogue recognizes that there is an indivisible solidarity between people and the world; it perceives reality not as a static entity but as an ever-changing process; and it is inseparable from action, and contextualized by time. Together, the five principles that form the epistemological basis of Freirean dialogue have guided how we constructed our research program to co-create knowledge with particular communities and across our research teams.

Freire’s ideas also shaped our sensitivity to “history from below.” Influenced by Marxist materialism, Freire [22] posited that we are all historical beings; hence, all human relationships are historical and therefore changeable. Thus, reflecting on our reality through dialogue has the potential to transform reality – the structures that determine people’s social locations and their trajectories. Engaging in dialogue reveals how people name their experiences (and how they self-identify), determine their strategies to produce or resist change (i.e., time allocation, individual and collective actions), and decide what is important for them and their loved ones (their values). From a Freirean perspective, love is paramount, because “No matter where the oppressed are found, the act of love is commitment to their cause of liberation. And because this commitment is loving, it is dialogical” [22. p. 89]. This means that Freirean-inspired researchers cannot interact with made-oppressed people merely to collect and extract data about their lived experiences by asking “objective” questions. Instead, we must share understandings, aspirations, and dreams with people and communities, requiring a deep commitment to listening and dialoguing with love.

Fundamentally, dialogue offers hope and a means of disrupting concrete reality by gathering with others and exchanging ideas. Dialogue needs time and the humility to listen very carefully, in order to understand the problems in the places where we are doing research. These activities, in turn, enable us to uncover what Freire calls the “thematic universe” – the set of themes and problems belonging to the daily life of a given group at a given historical moment [22, p.103]. Alongside universal and historical themes, each society is characterized by sui generis themes and situations particular to smaller units within each region or social group. Notwithstanding this diversity, each place remains interconnected with the broader societal context [22, p. 83].

Any attempt to dialogue with people without knowing, understanding, or seeing their thematic universe is like trying to read sheet music without understanding musical notation. To engage in meaningful dialogue, one must necessarily be immersed in that universe. Our DNA-informed research recognizes this first-hand: government responses to COVID-19 involved a series of recommendations that did not align with most people’s concrete realities, even if far less onerous for those most privileged (including the project PIs). As visibilized during the pandemic, consistent handwashing is impeded by limited water access; alcohol gel is prohibitively costly for people living on low incomes; and practicing social isolation in cramped homes is impossible. Such pandemic parameters and rules were designed outside the thematic universe of made-peripheral places and people –including various community-based research team members– with whom SMAPL has engaged, further exacerbating the social marginalization of these groups. For instance, in the Jardim Colombo favela, in Montréal’s most made-marginalized neighborhoods, and for MAWs living in bunkhouses on farms in Southwest Ontario, quarantine (or lockdown) measures were ineffective because of overcrowded living conditions. For Awajún communities, the *en masse* movement of Indigenous internal migrants from Peru’s coastal cities (where they were living, but lost their jobs and income amid the lockdown) to their geographically isolated communities in the Amazon region forced them to transgress quarantine rules.

Considering global health in dialogic terms has not only helped us re-think our own ways of doing research and nurturing relationships with communities, it has also enabled us to learn how communities, as policy shapers, organize themselves and enter into dialogue with decision-making authorities. The Montréal case study, for example, showed how creating a space for intergroup dialogue in an open and non-judgmental context made it possible to address under-vaccination (often combined with vaccination hesitancy and refusal) and contribute to the development of community-informed public health materials [36]. In Germany, meanwhile, communities organized their own vaccination campaigns in the absence of targeted state initiatives, and in the Peruvian Amazon, Indigenous health personnel obtained their vaccinations first as a way of convincing others to do the same.

## Grappling with collaborative research and global health: Structural approaches

As a global health research team, we have sought to address the structural dimensions of DNA via the project’s composition – involving researchers from two countries from the Global South and two from the Global North – as well as through the research team’s diversity in terms of gender, social class background, disciplinary specialty and expertise, and professional experience. Many of us hold credentials and resources that grant social recognition, authority, and power over others within academia, in society, and in the places where our research has taken place. The most privileged among us are faculty members; many are trainees pursuing academic degrees (several of whom are newly-minted professors). Only a few team researchers (none of the project PIs) are members of the communities with whom we are working (see list of additional team members at the end of the essay).

In our bimonthly all-country online meetings and at in-person sessions, team members are able to express themselves in the language of their choice, and simultaneous translation is carried out by polyglot colleagues (volunteers) or is professionally arranged (at some public events). This practice derives from our shared conviction that the dominant academic imperative of having to express oneself in English invariably generates uneven disadvantages and serves as a form of exercising and reinforcing the hegemony of the colonizer, which our research team seeks to resist.

Our reflection on inequalities within the project has included not only thinking about the relationships *between* the different teams based on their geographical location but also taking into account relations *within* each research team, especially in terms of redressing status differences. Intending to foster relations that are as equitable as possible, we have paid particular attention to ensuring a safe and welcoming space for trainees. These up-and-coming academics, who include community researchers in some of the settings, have been fully involved in discussions around project development, crafting and distribution of project outputs, and overall decision-making [37].

These internal dynamics have also helped build more solidarity-based relationships with the communities where we work. In Jardim Colombo, for example, learning from the community about its struggles during and beyond the pandemic involved numerous co-conceived and co-constructed multimedia strategies, including intensive knowledge exchange workshops and community oral history interviews. These activities spawned a host of materials including a comic book, videos, and website [11,38], which are now being used for educational outreach. Learning from and with the community has also involved providing scholarships to youth leaders from Jardim Colombo to become crucial team members, currently enabling them to pursue post-graduate studies based on the SMAPL research project.

Embodying DNA, the Bochum team has built on its decade-long experience of a City Lab, through which students, scholars, and community researchers engage in collaborative research, co-production of knowledge, and collective activism to contest and redress social and health inequalities. Each stage of the SMAPL project has been launched with joint knowledge-sharing sessions (e.g., on pandemics, local health needs, etc.), ensuring that community researchers contribute on equal footing. Community members have been actively involved in creating culturally sensitive research designs, co-analyzing research findings, co-presenting results to local stakeholders, as well as to university students, and co-authoring a paper [39]. Through this process, they have helped shape concrete interventions, including culturally-relevant vaccination campaigns, and have articulated demands on the local government to establish a community health center (currently underway), among other sustainable political, social, material, and capacity-building actions and community resources.

The Bochum team’s approach was shared horizontally with the Giessen team, and implemented locally thanks to close collaboration with a community center and a retired physician. Strikingly, at seminars organized by the University of Giessen’s Institute of the History, Theory and Ethics of Medicine, it was community researchers who taught medical students about health inequalities —rather than the other way around— thereby upending typical hierarchies.

In Peru’s Amazonas region, previous collaborations with health personnel, and a new one with a university student from an Awajún community who was interested in participating in the project and willing to introduce us to Indigenous communities and local organizations, were crucial during each phase of the research. This began with flexibility in defining objectives to reflect local priorities, and culminated with giving back the very stories that community members had shared, now in the form of accessible pamphlets and flyers – all strategies central to DNA-informed public health research [40]. Along the way, Indigenous health personnel and community members played a key role in the research process, the latter seeing themselves recognized through the community experiences recounted in the printed materials.

Although the Ontario team initially envisioned a community-driven project, it ultimately adopted a more “traditional” format of interviews of key players (both advocates and policymakers) at the behest of organizers supporting MAWs, whose time constraints, preoccupations, and pressing priorities we sought to respect. Responding to the needs of these organizers and of MAWs, the research team constructed a large database of activist-advocacy actions, government responses, and a visual timeline of events [41]. Through a “brokered dialogue” approach –as requested by MAW organizers and activists – the team has brought the voices of these players directly into interviews with politicians and other policymakers: on-the-ground concerns and demands have thus been directly channeled into the themes and questions put to policymakers.

The Québec team, meanwhile, pursued an activist research approach, whereby solidarity actions during the pandemic came first, and research came after. Between late 2020 and the summer of 2022, the lead researcher was involved in supporting the state vaccination campaign through almost daily dialogue with community groups and community leaders on the ground. The objective was to collectively develop initiatives and implement measures aimed at reducing barriers to vaccination—whether geographic, economic, or cultural. Socio-historical investigation of community-led interventions followed subsequently. This involved interviews, ethnography, and archival research, as well as the preparation of a paper co-analyzed and co-written with community actors from the Immigrant Workers Centre [42]. Dialogue, trust, and previous involvement in the community response to the pandemic enabled the collective writing of this paper.

Through our shared commitment to DNA across diverse community-responsive avenues, our project is also seeking to advance health equity, both directly and indirectly. As outlined above, in some settings this is taking place through new or enhanced recognition of how communities can pressure policymakers to respond to their needs (Germany, Montréal). In other places, the emphasis on recognizing the power of collective organizing efforts and local practices before, during, and since the pandemic has enabled community-driven approaches to health equity (and demands thereof) to flourish in renewed ways (Brazil, Peru, Ontario).

The temporal dimensions of academic cooperation represent another important structural aspect of our research. We recognize the importance of valuing the time needed for listening, multilingual interaction, and reflection in terms of “slow research” [43]. Our all-team meetings include progress reports, conceptual and methodological discussions, collective analysis of preliminary results, presentations and debates on key relevant readings from various disciplinary and geographic research contexts, and mutual reflections and interchange of understandings of theories and concepts. As well, during these meetings we plan in-person annual/biannual all-team gatherings, trainee exchanges, and website development, and share updates on projected writing, audio-visual, and other knowledge mobilization materials and events.

Meeting regularly has also supported our epistemological justice and equity aims, making space for, indeed encouraging, reflexivity, critical thinking, and debate. This space was particularly conducive for conversations about the significance of foregrounding scholarly contributions from the South and from feminist, anti-racist, Indigenous, anti-classist, anti-capitalist, and other counter-hegemonic epistemologies that we individually and collectively brought to the table as we each grounded our positionality in the context of DNA.

To provide just one illustration, many academics, especially in the Global North, have adopted the term “activist” as an umbrella concept referring to community leaders and sometimes local partners (plus scholar-activists) involved in research. However, through collective discussions regarding our respective understandings of the concept and experiences of activism, we were confronted with the fact, signaled by community members who are part of the research team, that most people in the communities with whom we have worked – those who actively tried to alleviate and contest the effects of the pandemic – do not identify as activists per se.

Far from being a purely linguistic matter, this interchange also reflects the conflict between activism and community organizing in contexts of poverty, as elucidated through Freire’s insights. Activism, when disconnected from a solid theoretical foundation, risks becoming disorganized and impulsive, with limited impact on structural transformations that address the root causes of poverty and oppression. Conversely, community organizing that relies solely on theory without engaging in the daily realities of local populations risks becoming verbalism, or offering solutions that fail to align with local needs.

Recognizing the value of this important discussion, we scheduled specific meetings to exchange perspectives and reflect collectively upon the contextual nuances of terminology used by and about those involved in community action during the COVID-19 pandemic (Table 2). These sometimes discordant dialogues drew from Freire’s argument that the power of naming is not (or ought not to be) an academic exercise, but rather one grounded in community understandings of people’s realities and aspirations for transformative possibilities through their engagement and community commitment.

**Table 2:** Diverse community understandings of the term “activists” and ways of naming people who actively sought to alleviate pandemic effects in their communities and place demands on policymaking.

Research Site	Community-level understandings and representations of “activists”	Self-identification of community members
Jardim Colombo, São Paulo	“Outside agitator” or others not connected in any real way to the community.	Engaged residents, artists, or community members.
Amazonas (Peru)	Political actors or NGO employees who have lost touch with local communities and follow their own interests.	Community leaders (including traditional healers), school teachers, or health providers.
Bochum	Researchers from the community.	Through community researchers’ involvement in SMAPL and other research projects, they have begun to see themselves as activists.
Giessen	Researchers from the community.	Before the project, they identified as ordinary residents; now they see themselves as community researchers and are recognized as such by others.
Ontario	Activist is often used as a label and is not necessarily viewed negatively. However, the term organizer is preferred.	Organizers, given their strategic role in movement-building and challenging asymmetrical power. Not all advocates are community-based, but all are solidaire/allied with MAWs and communities.
Montréal	Paid and unpaid individuals working at autonomous or self-governing community organizations or in formalized movements.	Community workers, community organizers, or militants (unpaid).

Following lengthy deliberations, we reached consensus around naming the people who are addressing urgent needs as “engaged community members,” while retaining the term activist in certain contexts. Yet another insight from this dialogue is that people’s (including our own) self-identification of roles in struggles and community activities is a process that takes time and reflexivity, and is not, or should not be, simply a passively-employed label. Moreover, because the term “activist” risks overemphasizing individuals over the collective, and because not all of those involved are community members per se, we have more recently updated our overarching characterization as “community organizer(s)” to more amply depict the various roles and commitments across SMAPL sites. In summary, this has been a multidirectional and ongoing learning process (à la DNA), wherein dominant homogenizing terms have not been superimposed on the project as a whole by the more “established” researchers´ preferred theoretical approaches; instead, ideas from many traditions have been considered in conjunction, even if at times held in tension.

This way of working has also contributed to, and intertwined with, our aforementioned epistemological discussions around centering concepts and theoretical approaches from the Global South, even as some critics might counter with a series of Northern theorists (see endnote iv). Shaped by these ideas, our methodological approach across research teams seeks to amplify the voices of the residents by telling the story of pandemic responses through their standpoint. This process of critical engagement has showcased the wide range of experiences in which made-marginalized communities in different Southern and Northern contexts responded to the COVID-19 pandemic. Although each SMAPL case study has adopted its own analytic approaches that incorporate local, regional, and international thinkers, the set of analytic dimensions we presented in Table 1 facilitates ongoing dialogue regarding the emerging findings and the echoes and reverberations of each case across the settings.

A prime illustration is that we have learned that all teams share a commitment to oral history approaches to documenting community residents’ perspectives on and experiences of the pandemic. Since the situated knowledge of a community’s struggle is contained in and expressed through stories, oral history methods allow us to elucidate this knowledge and find meaning in it within and across cases and disciplinary boundaries. In the context of a dialogic and counter-hegemonic approach, our focus on “history of the present” and on diverse subaltern recollections engenders reconstruction of the collective memory of local experiences of pandemic resistance and its policy repercussions [44–46].

This has further enabled us to contest the persistence of colonial practices in the field of global health, as represented by academic literature’s prevailing biomedical and technical approaches to depicting and explaining COVID-19, its emergence, and consequences. Each of our project’s case studies, inspired by Freirean action research, was constructed without the imposition of a research design by researchers from the North (thus, as discussed at the outset, differing markedly from many North-South global health research “partnerships,” which typically expect Southern partners to “follow”).

In pursuing these practices, we are committed to DNA across all dimensions of the research process. Emphasizing the redistribution of power and resources, our approach to global health seeks to address health inequities within and between places and people, *and* the underlying drivers of these inequities, highlighting the persistence of historically-shaped social, economic, and cultural structures as root causes of inequities wherever and whenever they occur. We recognize that this commitment resonates with the renewed calls and proposals for decolonizing global health that emerged simultaneously to the COVID-19 pandemic [47–49]. Indeed, initially we believed that there were prospects for this project to move towards a decolonial global health research praxis [50]. However, we did not reach consensus on what bona fide decolonization of global health entails, so we have continued to reflect on this matter from our respective positionalities and contexts, eschewing any overarching claims.

While some advocating for a decolonial paradigm shift [49,51] call for challenging social structures to embrace those who have long been dispossessed, left out, and politically unrepresented, we are concerned that most “decolonizing” global health efforts (will) remain at a symbolic level without dismantling the hegemonic power structures of financial decision-making, political agenda-setting, and the global capitalist-imperialist order writ large [48]. Underscoring this concern, Eve Tuck and K. Wayne Yang offer a powerful critique of the superficial appropriation of the concept of decolonization by researchers and academics in their influential article *Decolonization is Not a Metaphor* [52]. They argue that when decolonization is treated metaphorically it loses its radical and material essence, diverting attention from its core objectives: land restitution and the restoration of Indigenous sovereignty. With this critique in mind, we maintain our stance that framing our work as decolonial is problematic given the radical world-order transformation required to achieve this aspiration.

## Putting DNA into practice/action: Methodological matters

In the spirit of Freire’s praxis, and of upending convention, we now turn to how we have sought to operationalize DNA without actually presenting our research “findings.” In each setting, community members were approached to discuss their main concerns in the context of the COVID-19 pandemic: their interest in identifying and exploring particular themes was crucial to defining the focus of each case, along with the possibility of co-generating knowledge and resources useful for their own needs. The Jardim Colombo and Bochum projects embodied such a community-motored approach from start to finish. Elsewhere, the Ontario, Amazonas, and Giessen teams have used a combination of in-person meetings, email communications, and forums (town halls) to discuss knowledge mobilization strategies and receive suggestions and guidance. These teams are striving to provide targeted and community organizer-informed recommendations and demands to policymakers at various government levels; the Ontario team also intends to hold to account several agencies and ministries via a number of media strategies (e.g., press conferences, press releases, op-eds, blogs, etc.). In the Montréal case, reciprocity with the communities was conceived upstream, with action preceding research, not vice versa. In this sense, academic knowledge was put to the service of the communities from the start of activities and through continuous involvement responding to emerging needs, always acting with a view to social transformation.

To define our methods for each case study, we exchanged previous and ongoing research relationships and experiences in the field. The variety of methodological approaches developed across field sites to document local and context-specific responses to the pandemic are summarized in Table 3.

**Table 3:** DNA-driven methodological approaches developed by each field site.

Research Site	Participants and Researcher-Participants	Methods
Jardim Colombo, São Paulo	Community organizers (including several research team members) responsible for the response to the COVID-19 pandemic, community residents.	Exploratory interviews, community oral-history, participant observation, workshops for co-production of audio-visual content, and comic strips drawn by a favela artist.
Amazonas	Indigenous community leaders (Apus), community organizers, teachers, health providers, public officials^a^ at the local and regional level.	Exploratory interviews to define research questions, community oral-history, semi-structured interviews designed by the SMAPL team, observations, town halls and community events to present results to garner participants’ impressions, questions, and interpretations.
Bochum	Experienced community researchers (City Lab) from made-marginalized communities who co-organized health interventions with scholarly activists and civil society organizations during the COVID-19 pandemic.	Co-designed research questions, methods, interviews, and media projects with stakeholders, community researchers, and community members. Co-analysis sessions and co-presentation of research findings at town hall meetings, and joint advocacy to press policymakers to secure resources for community-based health care.
Giessen	Community members recruited and trained as community researchers, supported by experienced community researchers from Bochum.	Co-designed research questions and interviews with community researchers, community members, and local stakeholders; joint presentations of findings at town hall meeting and community events.
Ontario	Community organizers, activists, advocates, policymakers, activist researchers, journalists.	Semi-structured interviews designed by the SMAPL team, with questions raised in organizer interviews shaping interview protocols for policymakers. Reviews of social media and news outlets. Historical chronologies of key advocacy groups. Town Hall to share summaries and results with interviewees and other organizers, to garner their impressions, questions, and interpretations.
Montréal	Workers, organizers, activists, community leaders, project staff members (practitioners, researchers, and communication staff), government agency officials (public health authorities, Minister of Health and Social Services), philanthropic organizations.	Semi-structured interviews, participant-observation and ethnographic journaling, archival analysis, co-analysis and co-authoring with worker-organizers.

a.We use the term public “officials” to identify those who either work directly for municipal, regional, or national governments or who have other kinds of formal, often contractual, relationships with public health, education, or other local government social service providers – such as Bolsa Familia (Brazil), Juntos (Peru), or Quartiersmanagement (Germany) – including social programs’ local coordinators or supervisors, and health personnel.

Certainly the extent of community involvement in the research process has varied from case to case. In some instances, participants have taken on additional roles at specific moments, such as before research began, in order to (re)define relevant concepts, priorities, and outputs. In others, heightened community involvement has taken place in the middle, through engagement with preliminary results. Following Freire’s [21, pp. 37–41] steps toward a liberating methodology for research, we reflect here on how our practices embrace dialogue across the various stages of research. First, to maximize opportunities for horizontal contact with community members and to ensure mutual benefit for people participating in the project, the criteria utilized to identify and choose communities as sites for engagement were based on researchers’ prior relationships with community organizers, advocates, and those involved in community health (Montréal, Bochum, Giessen, Amazonas, Southwest Ontario) or with current and former students from those communities (Jardim Colombo and Amazonas).

Each SMAPL team began engaging in dialogue through an initial set of informal and formal interviews and group discussions with key interlocutors and, where possible, public officials, including subnational policymakers. Prior and new relationships with leaders of Indigenous organizations in Amazonas, and of well-known advocacy groups and public officials elsewhere, have increased our understanding of local pandemic responses. At times, this has allowed us to identify interlocutors from less expected quarters, such as Indigenous local health care workers in Amazonas and teachers and social workers in Giessen, who defied lockdown rules so as to continue supporting their communities. Other players (and actions) have included: public health workers building partnerships with community groups and involved in implementing vaccination campaigns in Montréal; community researchers in Bochum co-organizing a vaccination campaign; migrant worker advocates delivering food to MAWs on farms in Ontario; and community organizers distributing meals through an NGO in São Paulo.

Through open and in-depth conversations with these various actors, we explained the project’s overall objectives and provided the opportunity for collaboratively redefining the focus in each setting based on the degree of interest and involvement of community members. During exploratory visits and ongoing meetings alike, SMAPL teams across all settings remained committed to building and maintaining trustworthy relationships, making it clear that, if community members did not wish to be involved, the research would not continue at that site.

We also discussed which, and what kinds, of outputs would be useful and what community members’ roles would be, endeavoring to scaffold this work to communities’ envisioned social transformations and demands on policymakers. For example, communities in Jardim Colombo, Amazonas, and Germany decided to develop an array of digital (multimedia) materials. In preparing and issuing comic books, videos, and booklets, people were able to preserve their own experiences and histories, circulating these outputs within and beyond their communities as a form of recognition and reparation. In Giessen, Bochum, Ontario, and Québec, engaged community members requested various channels to bring their demands (or themselves) into contact with local stakeholders and politicians. In addition, several of the sites engaged in capacity-building and training strategies related to research, audio-video production, and policy advocacy. Together these efforts enhanced communities’ visibility and voices with the aim –if at times pursued circuitously– of mobilizing for health equity.

Following these extensive interactions, we took on Freire’s call to critically analyze participants’ discourse, accompanied by in-depth exploration of people’s perceptions expressed in interviews and discussions. Embracing Rivera Cusicanqui’s challenge to overcome researchers’ desire to seek synthesis (often termed generalizability) has for us entailed co-constructing histories of community responses to the pandemic. In doing so, we have sought to resist dominant approaches to comparative research. Instead, our collective examination of the six cases aims to fashion common elements of dialogue and to open pathways of interplay and mutual learning from the range of experiences and ways of engaging with policy responses across our various locales.

## Conclusion: Transforming global health research is/as a process

In describing our transnational research practices and our conceptualization of global health research from a dialogic perspective, this essay has sought to expand the horizon of possible practices that can be concretely implemented – here and now – in global health research projects. Our DNA-based global health prism has actively engaged communities as narrators of their own histories and as crucial and valid policymaking participants. In order to foster co-construction, via written and video documentation, of community experiences and memories, each team has organized workshops and public forums, facilitating shared processes of data analysis and narrative development.

At the heart of community mobilization is a concerted effort to challenge labor exploitation and the power and resource asymmetries marking the contemporary capitalist-imperialist world system. Cognizant that these asymmetries also permeate the research world, we do not claim to follow a community-based participatory approach per se. Instead, evoking Freire, our teams’ faith in, and hope for, the prospect of social justice struggles resisting and transforming the dominant hegemonic world order undergirds our methodological approach and commitment.

Notwithstanding these shared aspirations, implementing counter-hegemonic frameworks of knowledge production and reflexive dialogue has proven challenging at both ends of the research endeavor. At one end, grant funding, academic exigencies, and formal objectives that frame researchers’ work (and funders’ expectations) can limit the flexibility and horizontality envisioned by DNA. Despite these constraints, the five funding agencies that collaboratively supported this project were enthusiastic (ours was among just 6% of applications funded); future funding, however, remains a question mark. It may well be that the Trans-Atlantic Platform’s pandemic-era “Recovery, Renewal, and Resilience” initiative was a singular opportunity that may be difficult to revive or repeat, absent a DNA-type transformation in the global health research funding scenario, which is increasingly characterized by “evidence-based” commodification, commercialization, and privatization.

At the other end of the research dynamic, working with distinct communities according to our shared approach requires negotiating diverse expectations, needs, and temporalities while co-producing research in dialogue. This means acknowledging place- and community-specific differences and balancing multiple and competing priorities (e.g., pressing community concerns; grant-funding deadlines; team members’ individual educational, work, and scholarly goals; and ongoing and long-term commitments with communities).

Other challenges have to do with the *hows* of DNA research, such as engaging in slow research as a strategy to improve our methodology, even as the limited time frame for most research projects clashes with DNA. Further issues relate to the need for numerous (and time-consuming) research ethics board amendments deriving from our desire to respond to community preferences as they emerged and, of course, to academic (and funders’) expectations. We have also grappled with expectations regarding comparability, requiring considerable creativity in identifying ways of sidestepping formal comparisons to avoid homogenizing and totalizing our findings, an endeavor that, thankfully, is addressed through Freirean dialogue.

Admittedly, the very process of collectively conceiving of and writing this piece involved differences among the authors regarding theoretical referents and the modes of production and research of each team. Disagreements emerged, for example, concerning the criteria for deciding on key works for framing our research, e.g., whether it was essential to consider the ethical conduct of living authors to whom we refer, above and beyond the importance and relevance of their conceptual and theoretical contributions. Notably, the friction around this question did not fall strictly along South-North lines but also within each team.

Finally, we emphasize that the vision expressed here represents a multilingual collective with participants from four countries and spanning six languages. This piece was generated by many hands, which sought to confront and transcend the linguistic barrier of the English language as the standard for most scientific publications. The need for a final review in English has modified the original form of expression of the majority of authors. To address and provide accountability regarding this matter, we consulted extensively with the co-authors who are most proficient in English, and finalized this version jointly. Through this collaborative approach of shared dialogue and co-construction of knowledge, we have sought to heed DNA approaches not only via our research but also through our writing.

This piece thus reveals the challenges presented through/out the research process and the ways in which we are constantly (re-)inventing to continue our collective efforts. Because it is, or should be, in our very DNA (in its molecular biology sense) as global health justice researchers and practitioners to use DNA (that is, being true to the definition elaborated above: dialogical and non-extractivist approaches), we hope to sow seeds for what ought to be global health’s modus operandi and collective goal: the defense of health, understood in its broadest context, as a fundamental right for all.

**Box 2. Endnotes**

- We employ the term “made-marginalized,” “made-vulnerable,” and “made-peripheral” in lieu of “marginalized,” “vulnerable,” etc. so that the descriptor is not naturalized/personified but rather indicates ongoing processes of oppression and structural power asymmetries [4].
- We do not argue that decentralized policymaking is better at addressing local realities – only that it is a jurisdictional reality. We have found that power asymmetries, together with state surveillance and repression, are often reproduced at decentralized levels. Citing two Canadian pandemic examples, in Ontario, organizers, together with supportive media, visibilized how MAWs’ lack of residency and citizenship pathways, and their contracts tied to specific farm owners, enabled workplace abuses long overlooked and tolerated by provincial authorities. In Montréal, curfews particularly jeopardized people with precarious immigration status, who were under heightened surveillance when their work schedules required them to commute outside curfew hours. The Immigrant Workers Centre documented this problem, advocated for policy changes, and raised awareness in the media.
- We define Global South not purely geographically, but also in terms of “peoples and places that, historically and contemporaneously, have been made-marginalized via imperialism, capitalism, extractivism, militarism, and colonialism, wherever they occur” [9]. In turn, we define Global North as “a stand-in for the powerful actors, governments, corporations, foundations, and other financial and political elites that create and sustain the oppressive world order” (ibid). As such, our project arguably focuses upon the Global South, in the sense that the German and Canadian communities with whom we are working are comprised of migrants made-marginalized by imperialist regimes and logics, past and present.
- Research articles led by Global South scholars are often expected to use, cite, and pay their dues to Global North concepts and theorists, especially those who purport expertise in the South. We reject this approach: after all, every theorist is influenced by multiple other thinkers, although this is usually portrayed in exclusively North-South diffusionist terms. Moreover, the purpose of this article is not to present a genealogy of which/whose ideas are shaped by what/whom, but rather to harness the insights of certain notable thinkers from the South for global health research that emphasizes South-North learning. To the extent possible, we have also prioritized thinkers whose ethical practices, as far as we are aware, do not contradict the ethos/spirit of our research and this article.
